# Histological findings of sperm storage in green turtle (*Chelonia mydas*) oviduct

**DOI:** 10.1038/s41598-021-98917-6

**Published:** 2021-09-30

**Authors:** Chiyo Kitayama, Jumpei Tomiyasu, Hiroki Bochimoto, Satomi Kondo, Kazuyuki Tokuda, Ryuta Ogawa, Saki Okubo, Daisuke Kondoh

**Affiliations:** 1 Everlasting Nature of Asia (ELNA), Yokohama, Kanagawa 221-0822 Japan; 2 Department of Biodiversity Protection, Institute of Animal Reproduction and Food Research, Polish Academy of Sciences, 10-748 Olsztyn, Poland; 3 Division of Aerospace Medicine, Department of Cell Physiology, The Jikei University School of Medicine, Minato-ku, Tokyo, 105-8461 Japan; 4 Everlasting Nature of Asia (ELNA), Ogasawara Marine Center, Tokyo, 100-2101 Japan; 5 Laboratory of Veterinary Anatomy, Department of Veterinary Medicine, Obihiro University of Agriculture and Veterinary Medicine, Nishi 2-11 Inada-cho, Obihiro, Hokkaido 080-8555 Japan

**Keywords:** Marine biology, Electron microscopy, Ecology, Conservation biology

## Abstract

Green turtles (*Chelonia mydas*) are seasonal breeders with a time lag between mating and nesting periods. We therefore investigated whether female turtles store sperm like some other animals by histologically and ultrastructurally analyzing oviducts collected from three mature female free-ranging green turtles during the breeding season in the Ogasawara Islands, Japan. The oviduct comprised an infundibulum, magnum, isthmus, uterus, and vagina. Sperm was found in the isthmus of all turtles examined. Some spermatozoa were found in the duct and acini of glands in the isthmus of two turtles with oviducts containing eggs, and a few were also located in the transition area between the uterus and vagina of one of the turtles. On the other hand, we also found abundant spermatozoa on the luminal surface of the isthmus of one turtle captured during mating. In most reptiles, fertilization occurs in the infundibulum or albumen region, and thus the isthmus near those areas might be suitable for storing sperm in female turtles.

## Introduction

Green turtles (*Chelonia mydas*) are seasonal breeders^1^. The Ogasawara Islands comprise the largest rookery of this species in the northwest Pacific area^2–4^. Mature green turtles temporally migrate from their main feeding habitats in coastal areas of Honshu (the main island of Japan), to the Ogasawara Islands to mate^5,6^. Thereafter, male turtles gradually move back to their feeding habitats, whereas females remain and nest in the islands.

Considering these differences in the reproductive migration habits of male and female green turtles, asynchrony seems to occur between mating and nesting. Female green turtles can lay a clutch of eggs ~ 5 weeks after mating in a captive population^7^, but we previously found a 2–3 months lag between mating and nesting peaks in free-ranging green turtles^4^. The nesting season starts in early May and continues to late September in the Ogasawara population, whereas mating occurs mainly from March to May^4^. Furthermore, in a captive population, successive clutches demonstrate high fertility although the mating was only observed before the first clutch was laid^8^. These findings speculate that sperm storage in female green turtles.

Sperm storage is reported in female oviducts of some mammals, birds, amphibians, and reptiles, including other turtle species^9–12^. In these species, sperm stored in the oviduct can remain viable for long periods, which ensures fertilization despite asynchrony between insemination and ovulation^13,14^. The oviduct comprises an infundibulum, magnum (uterine tube), isthmus, uterus, and vagina^15^, and the location of sperm stored within the oviduct varies according to species. For example, sperm storage organ of neotropical rattle snakes (*Crotalus durissus terrificus*) is uterus^16^, and that of ground skinks (*Scincella laterale*) is a posterior vagina^17^. In some birds, the specialized glands for sperm storage are confirmed in vagina-uterus junction^18,19^, and the albumen glands (isthmus) in some turtle species^9,10,20,21^. Among sea turtles, spermatozoa stored in the oviduct was found from a single female of the olive ridley sea turtle (*Lepidochelys olivacea*)^10^, but this finding was a part of a study of many turtle species that analyzed the caudal 2 cm of the egg albumen-secreting region. Thus, more detailed evaluation is required to clarify how spermatozoa are stored in the oviduct of sea turtles, including green turtles.

Although the population of green turtles in the Ogasawara Islands is increasing^4^, the species is still listed in the International Union for Conservation of Nature (IUCN) Red List as endangered^22^. Therefore, conservation of this turtle in situ and ex situ is urgent. Sea turtles play important roles as prey, consumers, competitors, and hosts in marine ecosystems^23^. Understanding the reproductive mechanism that ensures fertilization in this endangered species is an important key to developing efficient strategies for its conservation. Here, we histologically and ultrastructurally analyzed whole oviducts collected from free-ranging, female green turtles in the Ogasawara Islands.

## Materials and methods

### Animals

Oviducts were collected from three dead mature female green turtles that were harvested and slaughtered as food by following Fisheries Adjustment Regulations of Tokyo which is strictly regulated under the permission of the governor of Tokyo, in Chichi-jima, Ogasawara Islands during the 2020 mating season between March and April. The Animal Care and Use Committee of Obihiro University of Agriculture and Veterinary Medicine approved the experimental protocol (Approval no: 21–27).

After the harvesting, straight carapace length, straight carapace width and body weight were measured in each turtle. Table 1 reports the physical data and harvested conditions of the animals. We dissected oviducts from the distal part of the vagina to the infundibulum of one turtle (ID: 035) and from the middle of the uterus to the infundibulum of two others (ID: 024 and 028), immediately after slaughter and stored them in 10% formalin. Moreover, as a reference, to check the condition of sperm maintenance in male turtles, we also collected epididymis from one adult male and observed it using a scanning electron microscopy.

**Table 1:** Harvested animals.

ID	SCL (cm)	SCW (cm)	BW (kg)	Capture status	Sampled oviduct
024	99.8	78.2	142.0	Single	Unknown
028	108.6	80.6	177.0	Mated	Right
035	94.0	74.8	129.0	Single	Right

*SCL* straight carapace length, *SCW* straight carapace width, *BW* body weight.

### Histological analyses

We dissected and embedded 29 parts of the oviduct of one turtle (ID: 035) in paraffin, after fixing. The sampled area comprised four from within the infundibulum (fimbria, and distal, middle, and proximal tubular areas), 17 at equal distance in the magnum, two in the isthmus (distal and proximal areas), three in the uterus (distal, middle and proximal areas), and three areas around the border between the uterus and vagina (proximal end of the uterus, transition area, and distal end of the vagina). The oviducts of the other two turtles (ID: 024 and 028) were dissected into 12 parts, comprising four in the infundibulum, three in the magnum (distal, middle, and proximal areas), two in the isthmus, and three in the uterus. Embedded samples were sliced into 5-μm-thick sections, deparaffinized, stained with hematoxylin–eosin, and assessed using a Microphot-FX microscope equipped with a Digital Sight DS-5M camera (both from Nikon Corp., Tokyo, Japan).

### Scanning electron microscopy

The formalin-fixed oviducts of two turtles (ID: 024 and 035) were further analyzed by scanning electron microscopy (SEM). The formalin-fixed samples were additionally fixed in 2% glutaraldehyde, cut into small pieces, washed thoroughly with 0.1 M phosphate buffer (PB; pH 7.4) then immersed in 1% tannic acid in PB for 2 h at 4 °C. The samples were washed with 0.1 M PB, immersed in 1% osmium tetroxide in PB for 2 h at 4 °C, dehydrated in an ascending series of ethanol (70%, 80%, 90%, 95%, and 100%) for 10 min each. The samples in 100% ethanol were then frozen in deeply chilled mortal with liquid nitrogen and cracked into particles with a single-edged razor blade and a hammer. After cracking, the samples were thawed in 100% ethanol, then transferred into t-butyl alcohol, and lyophilized in a VFD-21S freeze dryer (Vacuum Device Inc., Mito, Japan). The dried specimens were mounted on aluminum plates, coated with osmium using an HPC-1SW plasma device (Vacuum Device Inc, Mito, Japan), and examined by scanning electron microscopy (SEM) using a field emission Regulus 8100 SE microscope (Hitachi High-Tech, Tokyo, Japan) in SE mode. In our figures, sperms are colored using a Microsoft Power-Point software.

## Results

### Morphological features of the oviduct

**Figure 1:**
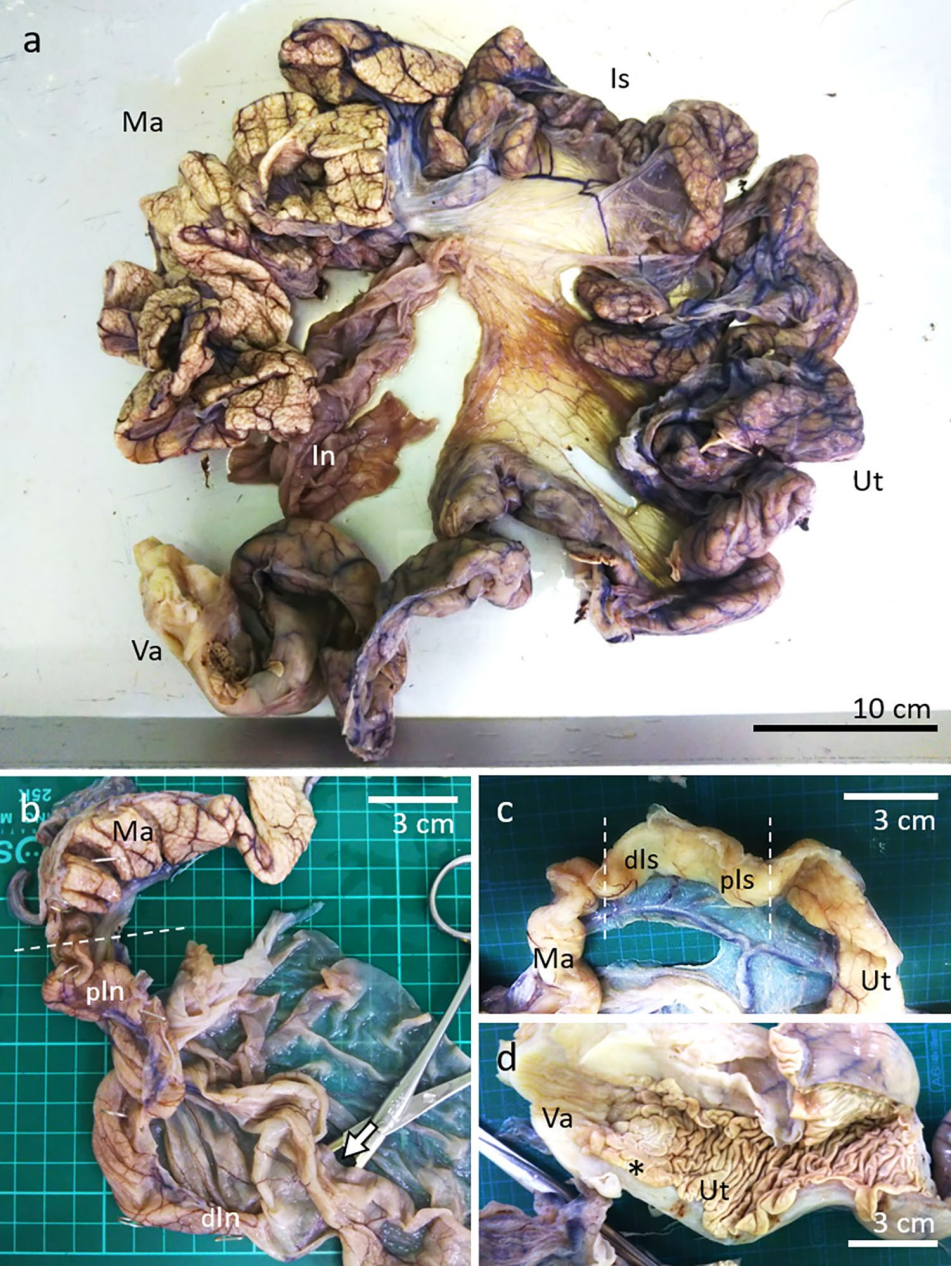
Morphological features of oviduct in green turtle (ID-035). (**a**) Image of entire oviduct from distal vagina to infundibulum. *In* infundibulum, *Is* isthmus, *Ma* magnum, *Ut* uterus, *Va* vagina. (**b**) High magnification of image near distal end of oviduct. Proximal (pIn) and distal (dIn) portions of infundibulum correspond to those in Fig. 2. Arrow, oviduct opening to abdominal cavity; *Ma* magnum. (**c**) High magnified image of transition area from Ut to Ma. Proximal (pIs) and distal (dIs) portions of isthmus correspond to those in Fig. 2. (**d**) *Internal views of transition area, correspond to Figs. 3 and 4, from (Va) to (Ut). Bars 10 (**a**) and 3 (**b–d**) cm.

The oviduct of turtle ID-035 was macroscopically divided into the infundibulum, magnum, isthmus, uterus, and vagina (Fig. 1a). The distal end of the infundibulum formed a fimbria, and the proximal portion became a thin-walled tubular structure that connected to the magnum (Fig. 1b). The proximal portion of the magnum transitioned to a thin-walled isthmus, followed by the uterus (Fig. 1c). The isthmus of green turtles was ~ 5 cm, but it was distinguishable from the magnum and uterus due to the difference in wall thickness (Fig. 1c). The thickness of the uterine wall was similar to that of the magnum and was continuous with the thick-walled vagina. The lumen of the uterus and vagina possessed large folds and slight longitudinal grooves, respectively (Fig. 1d). Mottled grooves in the narrow region between the uterus and vagina (Fig. 1d) were defined as a transition area between these two parts.

### Histological features of each part of the oviduct

**Figure 2:**
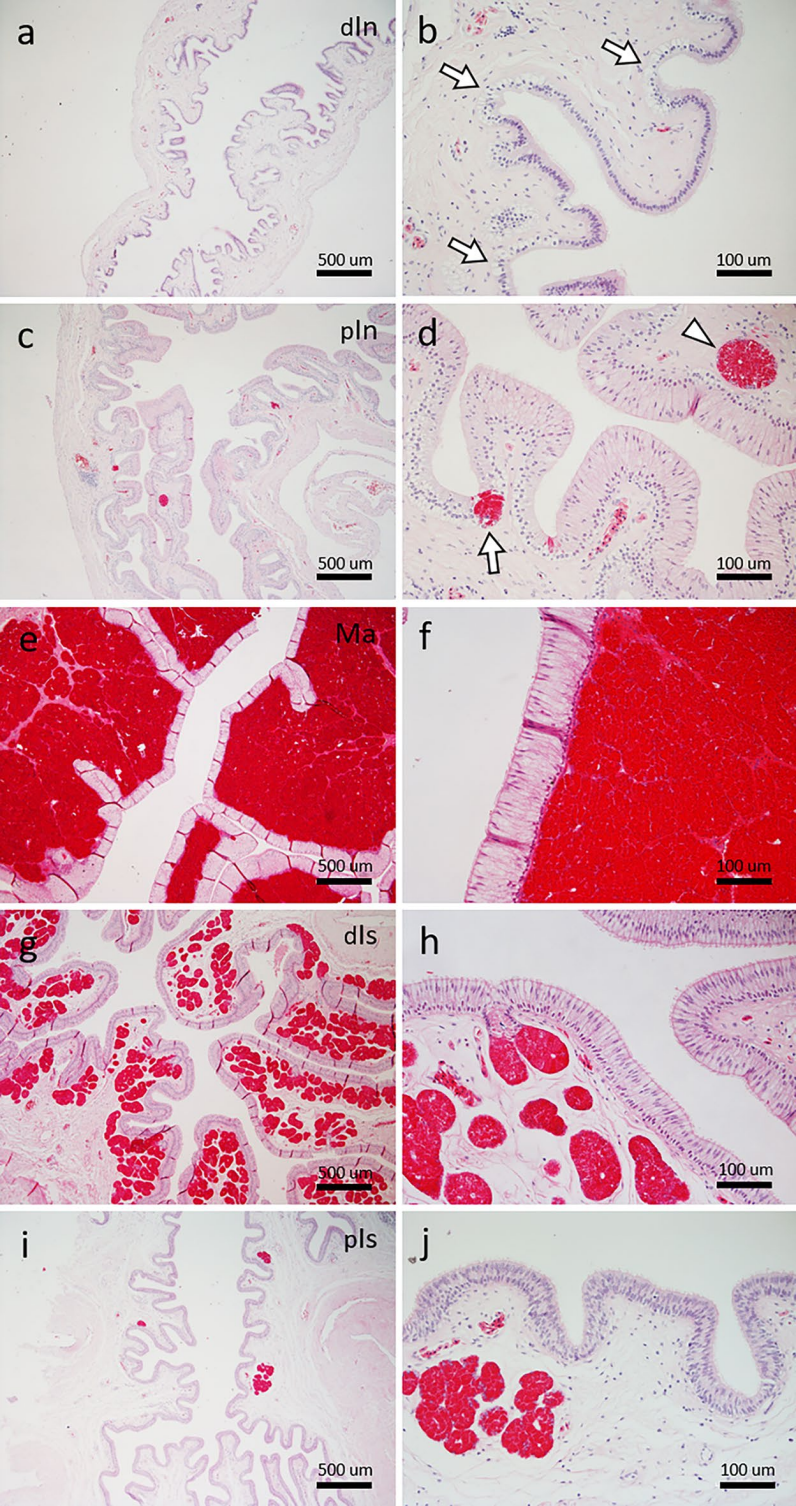
Histological features of oviduct from infundibulum to isthmus in green turtle (ID-035). (**a,b**) Distal portion of infundibulum (dIn). Arrows indicate interepithelial multicellular glands without eosinophilic granules. (**c,d**) Proximal portion of infundibulum (pIn). Arrow and arrowhead indicate eosinophilic interepithelial glands and eosinophilic acini of glands in lamina propria, respectively. (**e,f**) Magnum (Ma). Lamina propria contains eosinophilic glands. (**g,h**) Distal portion of isthmus (dIs). Eosinophilic glands are less abundant than those in magnum. (**i,j**) Proximal portion of isthmus (pIs) contains some eosinophilic glands. Bars 500 (**a,c,e,g,i**) and 100 (**b,d,f,h,j**) μm.

The distal portion of the infundibulum was lined by a simple columnar epithelium with non-eosinophilic, interepithelial multicellular glands located in epithelial depressions, but no gland structures were located within the lamina propria (Fig. 2a,b). The epithelium became pseudostratified with columnar cells in the proximal portion (Fig. 2c,d). Some interepithelial multicellular glands in the proximal infundibulum contained some eosinophilic granules, and eosinophilic acini of tubular glands of the infundibulum were also found in the lamina propria (Fig. 2c,d).

The magnum was lined by a pseudostratified epithelium like the proximal infundibulum, and eosinophilic glands in the lamina propria were evident throughout the magnum (Fig. 2e,f). The distal portion of the isthmus was also lined by a tall pseudostratified epithelium that contained many eosinophilic glands of the isthmus within the lamina propria (Fig. 2g,h). Columnar cells lining the isthmus became shorter in the proximal portion and the number of glands of the isthmus decreased (Fig. 2i,j).

**Figure 3:**
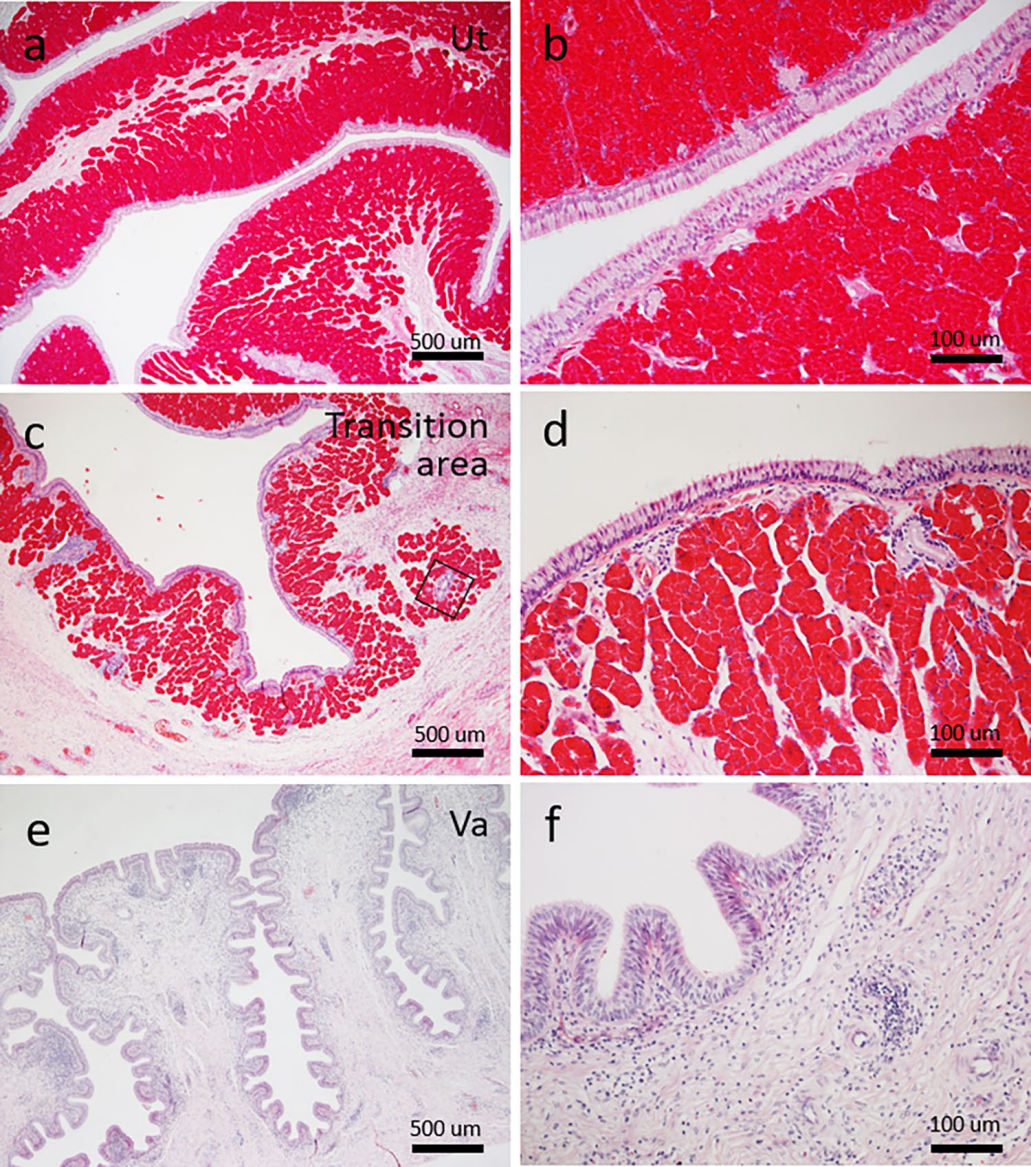
Histological features of oviduct from uterus to vagina in green turtle (ID-035). (**a,b**) Uterus (Ut). Lamina propria contains more eosinophilic glands than proximal portion of isthmus (also see also Fig. 2). (**c,d**) Transition area between uterus and vagina. Eosinophilic glands are sparser than those in uterus. Box (**c**) corresponds to Fig. 4c. (**e,f**) Vagina (Va). Lamina propria does not contain glands. Bars, 500 (**a,c,e**) and 100 (**b,d,f**) μm.

The uterus was lined by a short pseudostratified epithelium like the proximal isthmus, and the lamina propria throughout the uterus was full of eosinophilic uterine glands (Fig. 3a,b). The density and number of uterine glands decreased in the transition area between the uterus and vagina (Fig. 3c,d). The vagina was lined by pseudostratified epithelium, and gland structures were not found in the epithelium and lamina propria (Fig. 3e,f).

### Sperm in the oviduct of green turtles

**Figure 4:**
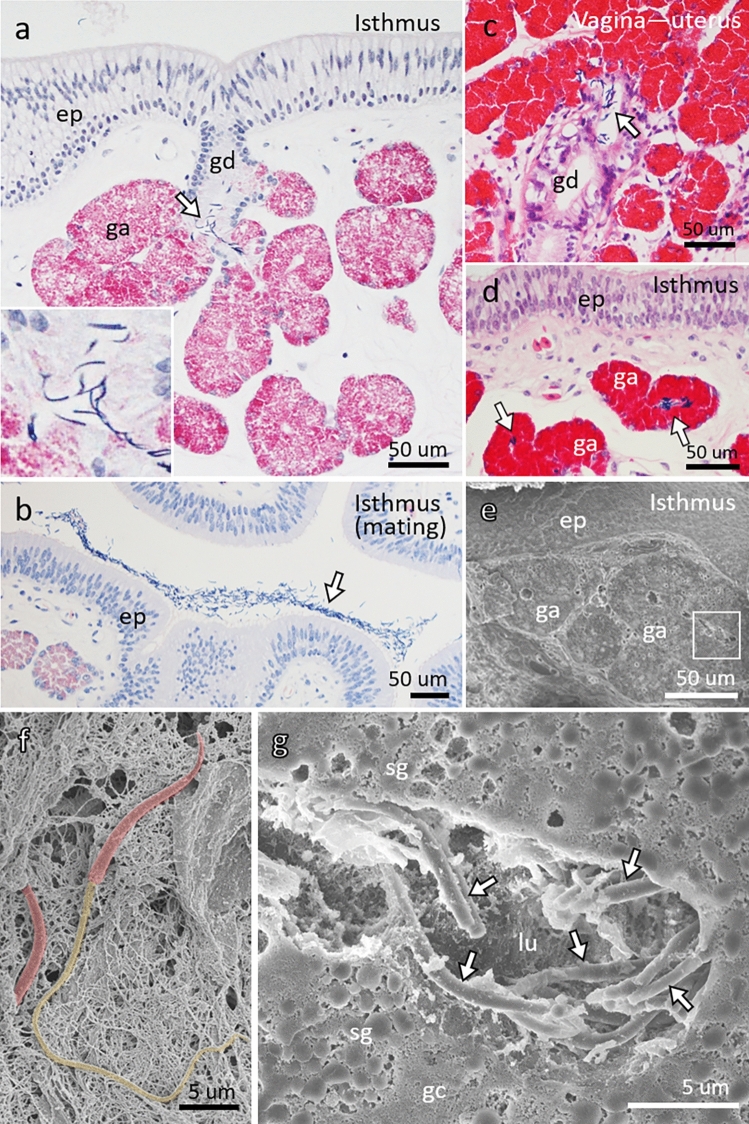
Sperm storage portion in oviduct of green turtle. (**a**) Sperm (arrow) in gland duct (gd) of isthmus in ID-024. Insert is higher magnification of sperm. ep, epithelium; ga, gland acini. (**b**) Sperm (arrow) in lumen of isthmus in ID-028 captured during mating. Spermatozoa are sparse in other parts of oviduct in this individual. (**c**) A few sperm (arrow) in gland ducts (gd) of transition area between uterus and vagina in ID-035. (**d**) Sperm (arrows) in gland acini (ga) of isthmus in ID-035. (**e**) Scanning electron microscopic (SEM) image corresponds to (**d**). Box corresponds to (**g**). (**f**) SEM image of spermatozoon in epididymis of a male green turtle. Red, head; yellow, tail. (**g**) SEM image of spermatozoa (arrows) in acini of isthmus glands. *gc* glandular cells, *lu* lumen of acini, *sg* secretory granules. Bars, 50 (**a–e**) and 5 (**f,g**) μm.

Table 2 summarizes the glands containing sperm in the turtle oviducts. Sperm was found in the isthmus of all three turtles examined. Some spermatozoa were located in the ducts and acini of the isthmus glands of two turtles (ID-035 and ID-024; Fig. 4a,d) that had eggs in their oviducts. In contrast, spermatozoa were found abundant in glands and the luminal surface of the isthmus in the turtle (ID-028) captured while mating (Fig. 4b). A few spermatozoa were also located in the transition area between the uterus and vagina (ID-035) (Fig. 4c). Sperm in the glands of the isthmus were 10–15 µm long, cylindrical, and attached to gland cells with secretory granules (Fig. 4e,f,g).

**Table 2:** Location of sperm in glands of oviducts collected from green turtles.

ID	Infundibulum	Magnum	Isthmus	Uterus	Transfer area (uterus–vagina)	Vagina
024	−	−	++	−	ND	ND
028	−	−	++ ^1^	−	ND	ND
035	−	−	++	−	+	−

Absent (−), some (+), and many (++) glands containing sperm.

*ND* not determined.

^1^Abundant sperm on epithelial surface.

## Discussion

The present study confirmed that sperm was stored in the isthmus of oviduct in all examined green turtles, like some other turtles studied^9,10,20,21^. Sperm is stored in glands of the isthmus that is located posterior to areas of albumen glands where albumen is secreted around the yolky ovum in turtles^9,10,20^. Although at this time it was not possible to assess the function of the isthmus glands for sperm storage, the isthmus might be suitable for storing sperm in the oviducts of female Testudines, including sea turtles because the isthmus is close to the infundibulum or albumen region where fertilization probably occurs in reptiles^15^.

Furthermore, we identified sperm in the transitional area between the uterus and vagina of one turtle (ID-035), indicating that this is another site of sperm storage in the green turtles, as well as stinkpot turtles^9^. In birds and American alligators (*Alligator mississippiensis*), the sperm is stored in both isthmuses, and largely vaginal-uterus junction^18,24^. After the oviposition of birds, the number of the glands with sperms in vaginal-uterus junction decreases, suggesting that the sperm is released from the vaginal-uterus junction for the fertilization^18^. Sperm seems to be excreted from a stored place by the physical stimulus through the movement of eggs in oviducts in birds and reptiles^9^. The sperm in the green turtle is also speculated to be stored transitionally in the vaginal-uterus junction and then released to the fertilization space. However, we could analyze the vaginal-uterus junction from only a single turtle, and thus further samples are needed to determine the correct role of this junction in the green turtle.

Glands in the isthmus of green turtles with and without sperm were histologically similar. Furthermore, the magnum and uterus also contained enlarged glands that were histologically similar to the glands in the isthmus. Palmer and Guillette (1988) found no specialized sperm storage glands in the isthmus and anterior uterus of the tortoise (*Gopherus polyphemus*)^20^, and ultrastructural analysis of oviducts from the isthmus and magnum of box turtles (*Terrapene carolina*) did not find any differences between these glands^25^. Therefore, all glands in the oviducts from the uterus to magnum seem to have similar characteristics and thus are thought to be potential storage sites for sperm. In fact, sperm is stored not only in the isthmus, but also in the uterus of Chinese soft-shelled turtles (*Trionyx sinensis*)^26^. Gist and Congdon (1998) postulated that the isthmus contains fewer and simpler submucosal glands and more developed ducts than the magnum and uterus, and that these ducts allow sperm better access^9^. Gist et al. (2008) also found the same characteristics in the area of sperm storage in American alligators^24^. The uterus and magnum of the green turtles contained only a few ducts, which might hinder sperm storage because of space limitations.

The time lag between mating and nesting in green turtles varies. Wood and Wood (1980) found that captive female green turtles laid their first clutch of eggs at an average of ~ 5 weeks after mating^7^, whereas the mating activity of free-ranging turtles in the Ogasawara Islands, peaks in the spring and the turtles start nesting during the summer, which is 2–3 months after mating activity peaks^4^. Individual sea turtles nest at least twice within one reproductive season, although the nesting interval varies depending on species and the area^27^. During the nesting season at the Ogasawara islands, female green turtles oviposit 4 times on average with a 2 week interval between each (data not shown), indicating that they can store spermatozoa for at least 4 to 5 months. Stored sperm enables female turtles to ensure that eggs are fertilized at some point in the future independently of males^28^. Considering the sea turtle reproductive ecology as described above, the duration of sperm storage in green turtles may be at least several months, like other turtles^29,30^. In alligators, the sperm storage is histologically observed only in breeding season^31^, whereas sperm storage in soft-shelled turtles is confirmed in a whole season^26^, suggesting that the duration of sperm storage differs among species. Female turtles should also be assessed during the non-mating season in future studies to confirm how long sperm can be stored.

The present study revealed the presence and location of spermatozoa stored within green turtle oviduct. However, understanding the interaction between stored spermatozoa and oviductal cells in green turtle remains a challenge. For example, the stored spermatozoa are specifically attached to ciliated cells in other turtles^26,32^, but is this also the case in green turtles? Does degradation of spermatozoa stored within unsuitable areas, which are reported in lizards^33^, occur in green turtles? To clarify the viability of sperms in green turtles in detail, further analyses using transmission electron microscope are required.
